# THE ROLE OF AGE AND COMORBIDITIES ON THE OUTCOME OF CONFIRMED CLINICALLY CRITICAL COVID-19 PATIENTS TREATED WITH REMDESIVIR AT INDONESIA’S NATIONAL REFERRAL HOSPITAL

**DOI:** 10.21010/Ajidv17i1.5

**Published:** 2022-12-22

**Authors:** USMAN Elly, KATAR Yusticia

**Affiliations:** Department of Pharmacology, Faculty of Medicine, Universitas Andalas, Padang, Indonesia

**Keywords:** Age, Comorbidities, COVID-19, Outcome, Remdesivir

## Abstract

**Background:**

There is currently no viable pharmaceutical therapy for COVID-19 illness that has been validated. The use of remdesivir is one of the medications for which there is no consistent evidence of a significant therapeutic benefit or a meaningful effect on survival.

**Aim:**

The aim of this study was to determine the role of age and comorbidities on the outcome of confirmed clinically critical COVID-19 patients treated with remdesivir at Indonesia’s National Referral Hospital.

**Methods:**

A retrospective cohort study was used in this study. The subjects in this study were confirmed clinically critical COVID-19 patients who were treated at Dr. M Djamil Hospital Padang, one of Indonesia’s national referral hospitals, from January 2 to June 30, 2021. The number of sample size in this study was 90 patients. The variables of this study were divided into three independent variables (age, comorbidities, and a number of comorbidities). A dependent variable was the outcome of confirmed clinically critical COVID-19 patients. The Chi-square test was performed in bivariate analysis, and the odds ratio was calculated. SPSS version 17.0 was used to analyze the data.

**Results:**

The results of this study found that there was an association between ages 50-59 years (OR = 10.23, 95% CI 1.89-55.53), 60-69 years (OR = 4.58, 95% CI 1.25-16.76), and > 70 years (OR = 1.91, 95% CI 1.38 -9.59), comorbid diabetes mellitus (OR = 9.78, 95% CI 1.23-77.66), the number of comorbid > 1 (OR = 10.97, 95% CI 2.19-54.96, and the number of comorbid 1 (OR = 5.69, 95% CI 1.59- 20.41) with the outcome of confirmed clinically critical COVID-19 patients treated with remdesivir.

**Conclusion:**

The significance of age and comorbidities on the outcome of COVID-19 patients treated with remdesivir at Indonesia’s national referral hospital was confirmed in this study. This study could assist in the management of patient therapy, potentially decreasing morbidity and even patient mortality.

## Introduction

Coronavirus disease 2019 (COVID-19) is a contagious illness. This disease has been declared a pandemic by the World Health Organization, which means that the disease’s spread is worldwide. There is currently no recognized pharmaceutical therapy for COVID-19 illness, and clinical trials are still ongoing (Li *et al.*, 2020; Lotfi *et al.*, 2020; Babaei *et al.*, 2021). Previous research found no indication of an effective therapy that reduced morbidity and mortality in COVID-19. COVID-19 treatment focuses on antivirals such as remdesivir, immunosuppressants, and immunomodulators because to the involvement of viral load and inflammatory response in the host (Pascarella *et al.*, 2020; Naik *et al.*, 2021).

Remdesivir is an adenosine nucleotide prodrug that is converted intracellularly to remdesivir triphosphate, which is the active substance (Kichloo *et al.*, 2021). Remdesivir’s active form binds to viral RNA-dependent RNA polymerase and inhibits viral replication. Remdesivir has shown *in vitro* action against SARS-CoV and MERS-CoV, with new *in vitro* results confirming its effectiveness against SARS-CoV-2 (Chalmers *et al.*, 2021).

Previous research has found no consistent evidence of a significant therapeutic benefit or a meaningful impact on survival with the usage of remdesivir (Liu *et al.*, 2020). Remdesivir was shown to be superior to placebo in terms of reducing recovery time in a randomized controlled experiment. In patients who were on mechanical ventilation, this favorable impact was not observed (Beigel *et al.*, 2020). Another trial indicated that 80% of patients were on mechanical ventilation, demonstrating the drug’s promise in critically ill patients (Pasquini *et al.*, 2020).

The aim of this study was to determine the role of age and comorbidities on the outcome of confirmed clinically critical COVID-19 patients treated with remdesivir at Indonesia’s National Referral Hospital.

### Materials and Methods

### Study design and research sample

A retrospective cohort study was used in this study. The subjects in this study were confirmed clinically critical COVID-19 patients who were treated at Dr M Djamil Hospital Padang, one of Indonesia’s national referral hospitals, from January 2 to June 30, 2021. The number of sample size in this study was 90 patients.

### Inclusion and exclusion criteria

COVID-19 patients were included in the study if the findings of an RT PCR/Molecular Rapid Test (TCM) SARS-CoV-2 collected from a nasal/nasopharyngeal swab were clinically critical. Patients with rapidly worsening acute respiratory distress syndrome (ARDS) or respiratory failure, as well as shock, encephalopathy, myocardial damage or heart failure, coagulopathy, acute renal impairment, multiple organ dysfunction, or other sepsis manifestations, are considered clinically critical. Patients above the age of 18 years who are being treated with remdesivir. Exclusion criteria were carried out on incomplete or unreadable patient medical record data.

### Operational definition

The variables of this study were divided into three independent variables, that is, age (<50 years, 50-59 years, 60-69 years, ≥ 70 years), comorbidities (hypertension, coronary arterial disease, diabetes mellitus, chronic lung disease, cerebrovascular, immunodeficiency, obesity, cancer), and number of comorbities (none, 1, ≥ 1). A dependent variable, that is, outcome of of confirmed clinically critical COVID-19 patients (death, life).

### Research ethics approval

This study passed the ethical review by the ethics commiittee of Dr M Djamil General Hospital, Padang, Indonesia (No. 27/ KEPK/ 2022).

## Data analysis

The results of the univariate analysis are reported as percentages and frequencies. The Chi-square test was performed in bivariate analysis, and the odds ratio was computed. If p<0.05, it is declared significant. SPSS version 17.0 was used to analyze the data.

## Results

Subject characteristics (Table 1).

Table 1 showed most subjects were 60-69 years old (36.7%), followed by 50-59 years (30.0%), <50 years (22.2%) and 70 years (11.1%). More than half of the subjects were male (57.8%). The most comorbidities were hypertension (33.3%), followed by diabetes mellitus (28.9%), chronic kidney disorders (17.8%), coronary arterial disease (13.3%), obesity (11.1%), cerebrovascular disease (3.3%), chronic lung disease (3.3%), malignancy (2.2%) and immunodeficiency (1.1%). Furthermore, number of patients with one comorbidity (35.6%), and patients with more than one comorbidity (32.2%).

**Table 1:** Subject characteristics

Characteristics	f (%)
**Age (years)**	
<50	20 (22.2)
50-59	27 (30.0)
60-69	33 (36.7)
**≥ 70**	10 (11.1)
**Sex**	
Male	52 (57.8)
Female	38 (42.2)
**Comorbidities**	
Cerebrovascular	3 (3.3)
Hypertension	30 (33.3)
Coronary arterial disease	12 (13.3)
Chronic lung disease	3 (3.3)
Cancer	2 (2.2)
Chronic kidney disease	16 (17.8)
Immunodeficiency	1 (1.1)
Diabetes mellitus	26 (28.9)
Obesity	10 (11.1)
**Number of comorbidities**	
None	29 (32.2)
1 comorbidity	32 (35.6)
>1 comorbidity	29 (32.2)

The role of age and comorbidities on the outcome of confirmed clinically critical COVID-19 patients treated with remdesivir at Indonesia’s national referral hospital (Table 2).

**Table 2:** The role of age and comorbidities on the outcome of confirmed clinically critical COVID-19 patients treated with remdesivir at Indonesia’s national referral hospital.

Variables	Outcome		p-value	OR (95% CI)

	Death (f/%) (n=71)	Life (f/%) (n=19)		
**Age (years)**			0.011*^a^	
<50	11 (55.0)	9 (45.0)		Ref
50-59	25 (92.6)	2 (7.4)		**10.23 (1.89-55.33)**
60-69	28 (84.8)	5 (15.2)		**4.58 (1.25-16.76)**
≥ 70	7 (70.0)	3 (30.0)		**1.91 (1.38-9.59)**
**Comorbidities**				
Cerebrovascular	3 (100.0)	0	n/a	n/a
Hypertension	26 (86.7)	4 (13.3)	0.315	2.17 (0.65-7.22)
Coronary arterial disease	11 (91.7)	1 (8.3)	0.448	3.30 (0.40-27.32)
Chronic lung disease	3 (100.0)	0	n/a	n/a
Cancer	2 (100.0)	0	n/a	n/a
Chronic kidney disease	14 (87.5)	2 (12.5)	0.507	2.09 (0.43-10.11)
Immunodeficiency	1 (100.0)	0	n/a	n/a
Diabetes mellitus	25 (96.2)	1 (3.8)	0.023*^a^	**9.78 (1.23-77.66)**
Obesity	9 (90.0)	1 (10.0)	0.682	2.61 (0.31-22.02)
**Number of comorbidities**			0.001*^a^	
None	16 (55.2)	13 (44.8)		Ref
1 comorbidity	28 (87.5)	4 (12.5)		**5.69 (1.59-20.41)**
>1 comorbidity	27 (93.1)	2 (6.9)		**10.97 (2.19-54.96)**

* p<0.05 considered significant; a, Chi-square test

Table 2 shows comorbid cerebrovascular, hypertension, coronary arterial disease, chronic lung disease, cancer, chronic kidney disease, immunodeficiency, and obesity had no association on the outcome of confirmed clinically critical COVID-19 patients treated with remdesivir (p>0.05). But, there was an association between age 50-59 years (OR = 10.23, 95% CI 1.89-55.53), 60-69 years (OR = 4.58, 95% CI 1.25-16.76), and > 70 years (OR = 1.91, 95% CI 1.38 -9.59), comorbid diabetes mellitus (OR = 9.78, 95% CI 1.23-77.66), the number of comorbids > 1 (OR = 10.97, 95% CI 2.19-54.96, and the number of comorbid 1 ((OR = 5.69, 95% CI 1.59- 20.41) with the outcome of confirmed clinically critical COVID-19 patients treated with remdesivir.

## Discussion

The results of this study found that there was an association between age 50-59 years (OR = 10.23, 95% CI 1.89-55.53), 60-69 years (OR = 4.58, 95% CI 1.25-16.76), and > 70 years (OR = 1.91, 95% CI 1.38 -9.59), comorbid diabetes mellitus (OR = 9.78, 95% CI 1.23-77.66), the number of comorbids > 1 (OR = 10.97, 95% CI 2.19-54.96, and the number of comorbid 1 (OR = 5.69, 95% CI 1.59- 20.41) with the outcome of confirmed clinically critical COVID-19 patients treated with remdesivir.

Remdesivir works by inhibiting the viral RNA polymerase that is dependent on viral RNA. Remdesivir has previously been shown to be effective in limiting viral replication and decreasing coronavirus-associated disease *in vivo*. It’s unclear how Remdesivir’s direct antiviral activity might be active throughout the immunopathogenic ARDS phase of COVID-19 disease, implying that off-target consequences could be blamed on the medicine (Nile *et al.*, 2020; Eastman *et al.*, 2020).

Despite severe illness (57% on mechanical ventilation) and severely (8% on extracorporeal membrane oxygenation), another trial in 53 patients from 9 countries getting remdesivir for 1 to 10 days demonstrated clinical improvement in 68 percent of patients, with just 15% exhibiting worsening with COVID-19. Interestingly, improvement was reported in 100% of patients with mild COVID-19 (not getting oxygen support or low-flow oxygen) and 71% of patients with severe COVID-19 (not receiving oxygen support or low-flow oxygen) (receiving high-flow oxygen support) (Singh *et al.*, 2020).

Because comorbidities frequently rise with age, COVID-19 is more severe in the elderly population among COVID-19 patients. The pathophysiological alterations that characterize the respiratory system are associated with worse outcomes as people age. Patients over the age of 80 who have been infected with COVID-19 have a higher chance of death than younger patients, according to existing epidemiological data (Perrotta *et al.*, 2020).

In a previous study that involved 80 patients with COVID-19 treated with remdesivir, patients who were younger at the time of discharge had a higher rate of clinical improvement than those who were older. The death rate in older patients is higher than in younger patients within 60 days after diagnosis of COVID-19 (Kanai *et al.*, 2021).

Patients infected with SARS-CoV-2 who were admitted to Pesaro Hospital’s ICU and given remdesivir medication were studied in previous investigations (Pasquini *et al.*, 2020). According to research, comorbidity is a factor that is significantly linked to a higher death rate. Hypertension (54.9%), diabetes mellitus (13.7%), ischemic heart disease (13.7%), chronic kidney failure (7.8%), chronic heart failure (7.8%), and chronic obstructive pulmonary disease (COPD) (7.8%) were the most prevalent comorbidities (Pasquini *et al.*, 2020). In this study, it was discovered that remdesivir medication was linked to a higher rate of survival (Pasquini *et al.*, 2021).

The body is under a lot of stress in individuals with comorbid conditions like diabetes and hypertension, and their immunity is usually low (Nindrea, 2022). Furthermore, a long-term history of diabetes and hypertension damages blood vessel structure and increases the risk of critical illness (Petrie *et al.*, 2018). Patients with chronic heart illness are more likely to become infected due to weakened heart function and inadequate immunity, putting them at risk of developing acute cardiovascular events and severe disease if infected with COVID-19 (Alqahtani *et al.*, 2022). Patients with a history of respiratory disease, such as COPD, have lower viral resistance and are more likely to develop acute respiratory distress syndrome (ARDS) (Saguil *et al.*, 2020). Diabetes, hypertension, cardiovascular disease, and respiratory disease are all risk factors for disease development (Zheng *et al.*, 2020; Afriani *et al.*, 2022).

This study’s main strength was being the first study to the role of age and comorbidities on the outcome of confirmed clinically critical COVID-19 patients treated with remdesivir at one of Indonesia’s national referral hospitals. Furthermore, our analysis contained several limitations. Because the data used in this analysis were retrospective, it was not possible to compare the findings to those of the comparison group. Data were gathered at a specific moment in time. However, our analysis contributes to the clinical data on the use of remdesivir in COVID-19 by presenting results of a cohort of COVID-19 patients treated with the drug in actual clinical settings in Indonesia.

The implications of the results of this study suggest that clinical research and trials involving a wider population are needed to determine the effectiveness of remdesivir. Remdesivir’s function in upfront remdesivir use in the general population based on age and comorbidities, as well as its effectiveness against other versions, are clinical questions that have not yet been resolved. This research may help with patient therapy management, perhaps lowering morbidity and even patient mortality.

## Conclusion

The significance of age and comorbidities on the outcome of COVID-19 patients treated with remdesivir at Indonesia’s national referral hospital was confirmed in this study. This study could assist in the management of patient therapy, potentially decreasing morbidity and even patient mortality.
